# Secondary-Sphere Effects in Molecular Electrocatalytic CO_2_ Reduction

**DOI:** 10.3389/fchem.2019.00397

**Published:** 2019-06-13

**Authors:** Asa W. Nichols, Charles W. Machan

**Affiliations:** Department of Chemistry, University of Virginia, Charlottesville, VA, United States

**Keywords:** inorganic, electrocatalysis, CO_2_, secondary-sphere, molecular

## Abstract

The generation of fuels and value-added chemicals from carbon dioxide (CO_2_) using electrocatalysis is a promising approach to the eventual large-scale utilization of intermittent renewable energy sources. To mediate kinetically and thermodynamically challenging transformations of CO_2_, early reports of molecular catalysts focused primarily on precious metal centers. However, through careful ligand design, earth-abundant first-row transition metals have also demonstrated activity and selectivity for electrocatalytic CO_2_ reduction. A particularly effective and promising approach for enhancement of reaction rates and efficiencies of molecular electrocatalysts for CO_2_ reduction is the modulation of the secondary coordination sphere of the active site. In practice, this has been achieved through the mimicry of enzyme structures: incorporating pendent Brønsted acid/base sites, charged residues, sterically hindered environments, and bimetallic active sites have all proved to be valid strategies for iterative optimization. Herein, the development of secondary-sphere strategies to facilitate rapid and selective CO_2_ reduction is reviewed with an in-depth examination of the classic [Fe(tetraphenylporphyrin)]^+^, [Ni(cyclam)]^2+^, Mn(bpy)(CO)_3_X, and Re(bpy)(CO)_3_X (X = solvent or halide) systems, including relevant highlights from other recently developed ligand platforms.

**GRAPHICAL ABSTRACT:**
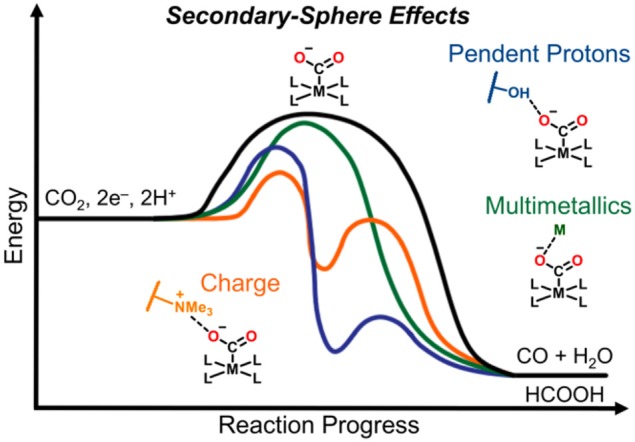
Secondary-sphere strategies known to improve catalytic selectivity and activity include pendent proton donors/shuttles in the form of Brønsted sites, charged moieties, sterically demanding functional groups, bimetallic active species, and stoichiometric participation of alkali and alkaline earth metal cations.

## Introduction

The development of scalable and cost-effective processes to store electrical energy in chemical bonds using CO_2_ as a primary feedstock remains a significant challenge for energy research (Centi and Perathoner, 2009; Senftle and Carter, 2017). Of particular interest are homogeneous catalysts for CO_2_ reduction, as their well-defined structures give chemists an opportunity to effectively characterize intermediates relevant to the operating mechanism and further optimize active catalyst families through iterative ligand design (Benson et al., 2009; Rountree et al., 2014). While the influence of different ligand types in the primary coordination sphere can be observed in the catalytic activity and selectivity of transition metals across the *d*-block towards CO_2_ reduction, more subtle effects—including rate enhancement and lowered overpotentials—can be obtained through modulation of the secondary coordination sphere within specific classes of metal complexes (Jiang et al., 2019). This has been particularly successful in the design of highly active and selective catalysts for CO_2_ reduction, directing a paradigmatic shift in the general understanding of “design principles” (Costentin et al., 2012a, 2014a,b; Sampson et al., 2014; Azcarate et al., 2016; Ngo et al., 2017).

While this review focuses on molecular catalysts which were specifically examined for electrochemical CO_2_ reduction, secondary-sphere effects have been successfully harnessed in related catalytic processes, including thermal CO_2_ hydrogenation (Himeda et al., 2004, 2005, 2007; Hull et al., 2012; Wang et al., 2012, 2013, 2014; Manaka et al., 2014; Suna et al., 2014; Cammarota et al., 2017), hydrogen evolution (Curtis et al., 2003; Henry et al., 2005, 2006; Wilson et al., 2006; Fraze et al., 2007; Jacobsen et al., 2007a,b; DuBois and DuBois, 2009; Gloaguen and Rauchfuss, 2009; Helm et al., 2011; Reback et al., 2013; Ginovska-Pangovska et al., 2014), hydrogen oxidation (Curtis et al., 2003; Henry et al., 2005, 2006; Wilson et al., 2006; Fraze et al., 2007; Jacobsen et al., 2007a,b; Dutta et al., 2013, 2014; Ginovska-Pangovska et al., 2014), formate oxidation (Galan et al., 2011, 2013; Seu et al., 2012), and oxygen reduction (Collman, 1977; Collman et al., 2004; Lewis and Tolman, 2004; Mirica et al., 2004; Fukuzumi, 2013; Ray et al., 2014; Fukuzumi et al., 2015; Nam, 2015; Sahu and Goldberg, 2016; Elwell et al., 2017; Hong et al., 2017; Sinha et al., 2019) reactions. In this review, we focus on how the mechanism of CO_2_ reduction relates to the type of secondary-sphere effects employed in molecular systems. Strategies discussed here which have been shown to increase catalytic activity and selectivity include pendent proton donors/shuttles in the form of Brønsted sites, charged moieties, sterically demanding functional groups, bimetallic active species, and stoichiometric participation of alkali and alkaline earth metal cations (Graphical Abstract).

To contextualize the motivation and principles, examples of secondary-sphere effects in enzymes which catalyze the interconversion of CO_2_ with either CO or formic acid are discussed. This overview is followed by a careful examination of secondary-sphere effects in several abiotic molecular electrocatalyst examples, beginning with the [Fe(tetraphenylporphyrin)]^+^ [Fe(TPP)]^+^ systems pioneered by Savéant, Robert, and Constentin, including a discussion of the effects of pendent proton source placement and the distance dependence of through-space effects induced by charged residues (Costentin et al., 2014a,b; Manbeck et al., 2015; Mohamed et al., 2015; Azcarate et al., 2016; Zahran et al., 2016; Margarit et al., 2018; Nichols et al., 2018b; Sinha and Warren, 2018). Next, M(bpy)(CO)_3_X catalysts (M = Mn or Re; X = solvent molecule or halide) (Wong et al., 1998; Bourrez et al., 2011; Smieja et al., 2013; Chabolla et al., 2014, 2017; Franco et al., 2014; Machan et al., 2014a, 2015, 2016; Riplinger et al., 2014; Agarwal et al., 2015; Manbeck et al., 2015; Riplinger and Carter, 2015; Machan and Kubiak, 2016; Ngo et al., 2017; Sahu et al., 2017; Sinha et al., 2019) in which steric parameters, pendent Lewis acid effects, and charged residues have been shown to be effective will be discussed. Finally, [Ni(cyclam)]^2+^ (cyclam = 1,4,8,11-tetraazacyclotetradecane), which contains pendent proton donors on the coordinating N atoms of the macrocycle, is discussed (Beley et al., 1984; Barefield et al., 1986; Balazs and Anson, 1993; Kelly et al., 1999; Froehlich and Kubiak, 2012; Song et al., 2014; Nichols and Chang, 2018). These systems are among the most highly studied in the field and representative of the progress that has been made in understanding how secondary-sphere coordination effects enhance molecular electrocatalysis. Additional discussions on emerging systems for CO_2_ reduction which utilize secondary-sphere effects are included to summarize some of the current work in the field.

## Enzymes for the Interconversion of CO_2_ and CO or Formic Acid

### Cu,Mo-Containing Carbon Monoxide Dehydrogenase (Cu,Mo-CODH)

**Figure 1:**
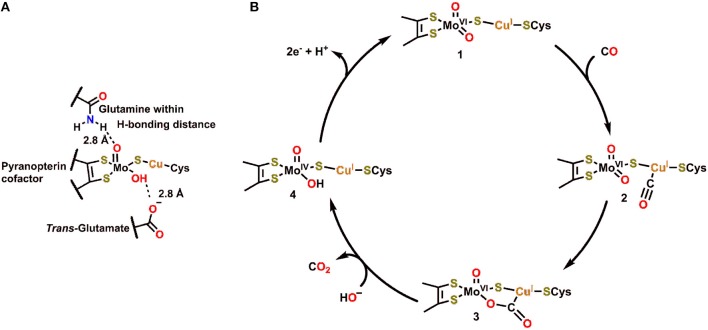
**(A)** Active site of Cu,Mo-CODH. **(B)** Proposed catalytic cycle for oxidation of CO by Cu,Mo-CODH (Dobbek et al., 2002; Kroneck and Torres, 2014).

The structure and function of Cu,Mo-CODH enzymes have been previously reviewed in great detail (Appel et al., 2013; Kroneck and Torres, 2014). Of this class of enzyme, the Cu,Mo-CODH in *O. carboxydovorans* has been the most extensively studied (Figure 1A) (Dobbek et al., 2001a, 2002; Hille, 2013). The active site of this enzyme contains Mo and Cu ions, which are bridged by a μ_2_-sulfido ligand. Mo is coordinated in a distorted square pyramidal fashion by an enedithiolate moiety (from the pyran ring found in the pyranopterin cofactor), a μ_2_-sulfido ligand, and an oxo/hydroxo ligand in the equatorial plane. The apical ligand is an oxo, with a glutamine residue within hydrogen-bonding distance and a glutamate residue in a *trans* position. A unique structural feature of Cu,Mo-CODH in comparison to other Mo-containing hydrogenases is its covalent linkage through a cysteine residue to the Cu ion, connecting the heterobimetallic active site to the large subunit of the CODH enzyme.

In the catalytic cycle for transformation of CO to CO_2_ by Cu,Mo-CODH, CO is proposed to initially bind to the Cu(I) ion **2**, followed by nucleophilic attack by the equatorial oxo ligand to generate the bridging μ_2_-η^2^-CO_2_ adduct **3** (Figure 1B) (Dobbek et al., 2002). This five membered metallacycle results from a redox-neutral reaction which maintains the Mo(VI) state (Figure 1B). Following rearrangement and net oxidation of CO, CO_2_ release occurs with hydroxide binding to produce the Mo(IV) species **4**. Subsequent 2e^−^ oxidation returns the Mo active site to the initial Mo(VI) oxidation state. Notably, the glutamine residue, which is in contact with the equatorial oxo ligand (Figure 1B), can act as a Brønsted base (proton acceptor) to regenerate the more nucleophilic oxo ligand when transitioning from **4** to **1**. If the reverse of this catalytic cycle is imagined, it is clear that a proton-donating residue would be beneficial for CO_2_ reduction and C–O bond cleavage to produce CO.

### Monofunctional Ni,Fe-Containing CODHs

**Figure 2:**
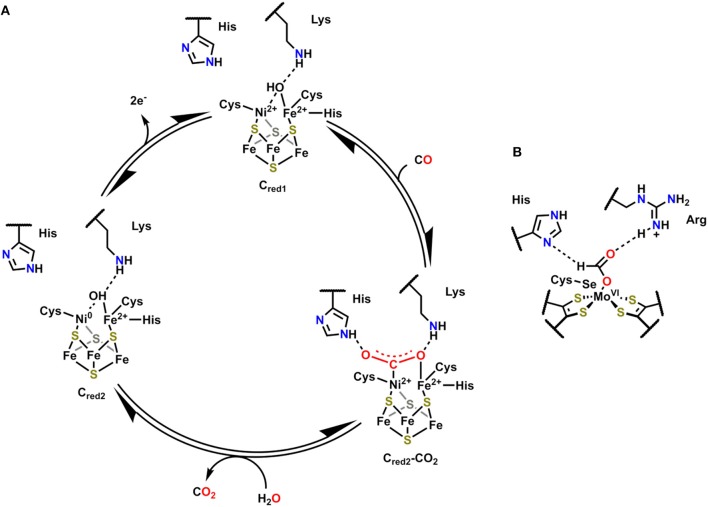
**(A)** Proposed mechanism for CO oxidation by Ni,Fe CODH (Jeoung and Dobbek, 2007). **(B)** Active site of FADH in *Escherichia coli* with bound formate substrate (Boyington et al., 1997).

The active site of Ni,Fe-containing CODHs, known as cluster C, is proposed to cycle between three separate redox states during catalytic CO oxidation (C_red1_, C_int_, and C_red2_; Figure 2) (Jeoung and Dobbek, 2007). The behavior and activity of cluster C from *Carboxydothermus hydrogenoformans* have been experimentally interrogated in these three different redox states utilizing chemical-reducing agents (Jeoung and Dobbek, 2007). The cofactor contains an Fe_4_Ni cluster bridged by sulfide ligands with a single Fe and Ni atom in the active site, which also contains histidine and lysine residues in close proximity to interact with active site-bound substrate molecules. In C_red1_, the active site contains Fe^2+^ and Ni^2+^ ions with an Fe-bound hydroxo ligand that is within hydrogen-bonding distance of the proximal lysine residue. Upon exposure to CO, the Fe-bound hydroxide is deprotonated, and the resultant oxo species can form a new C–O bond to generate the μ_2_-η^2^-CO_2_ adduct C_red2_-CO_2_, where CO_2_ is bound through C by Ni and O by Fe (Figure 2) (Jeoung and Dobbek, 2007). This μ_2_-η^2^-CO_2_ binding mode is stabilized by hydrogen-bonding interactions with the pendent histidine and lysine residues (Jeoung and Dobbek, 2007). A 2e^−^ oxidation with concomitant binding of an aquo ligand reduces the Ni^2+^ to Ni^0^ in C_red1_, triggering release of CO_2_. Oxidation of the reduced cofactor by 2e^−^ regenerates the C_red1_ catalytic resting state.

### Formic Acid Dehydrogenase

Formic acid dehydrogenase (FADH) enzymes catalyze the reversible 2e^−^/1H^+^ interconversion of CO_2_ and formate, another reaction of interest to CO_2_ utilization (Sawers, 1994). The active site of the FADH enzyme in *Escherichia coli* contains a single Mo(VI) atom coordinated to four S atoms from two *cis*-dithiolene moieties originating from the bis(molybdopterin guanine dinucleotide) cofactor, a hydroxyl ligand, and a SeCysteine (Boyington et al., 1997). The active site also contains histidine and arginine residues in a position to interact with activated Mo-bound intermediates (Figure 2B). The catalytic oxidation of formate begins by displacement of the hydroxyl ligand with an equivalent of formate, which binds as an η^1^-OCHO ligand; the η^1^*-O* binding mode is stabilized through hydrogen-bonding interactions between the arginine and histidine residues and the unbound H and O atoms of formate (Figure 2B). Subsequent oxidation and transfer of 2e^−^ from formate to the Mo center occurs with the release of CO_2_ and proton transfer to the SeCysteine residue. The Mo(IV) center can then be returned to the resting state through the loss of 2e^−^ as the histidine deprotonates the Mo-bound SeCysteine. Abiotic structural motifs similar to these three examples are discussed in the subsequent sections where either a) bimetallic active sites or b) secondary-sphere moieties are used to mediate electrochemical CO_2_ reduction.

### Benchmarking Molecular Electrocatalysts for CO_2_ Reduction

Electrochemical techniques can facilitate the determination of kinetic and thermodynamic parameters for both Faradaic and catalytic reactions. Cyclic voltammetry (CV), a nondestructive potentiostatic technique, is particularly informative. Indeed, the breadth and importance of rigorous CV analysis have been reviewed in great detail (Savéant, 2008, 2018; Costentin et al., 2012b; Costentin and Savéant, 2014, 2018a; Rountree et al., 2014). Experimental determination of fundamental reaction parameters is essential for comparing the effects of pendent functional groups on the activity of electrocatalysts.

The effective catalytic overpotential (η) is the difference between the standard potential of CO_2_ reduction ($E_{\text{CO2/CO}}^{0}$) and the potential at half catalytic current height (*E*_cat/2_) as described in Equation (1) and describes a thermodynamic quantity: the electrochemical energy beyond the standard potential which is required to drive a reaction of interest at an appreciable rate. We note that some prefer to define the overpotential term as *E*_CO2_−*E*_1/2_. We distinguish between these through the use of “effective catalytic overpotential” to describe the overpotential calculated utilizing *E*_cat/2_ (Appel and Helm, 2014).

(1) $$\begin{array}{r} \eta =E_{\text{CO2/CO}}^{0}-E_{\text{cat}/2} \end{array}$$

Another reaction parameter commonly measured through CV is the maximal turnover frequency (TOF_max_), which can also be described as the observed catalytic rate constant (*k*_obs_) with units of s^−1^ (Costentin and Savéant, 2014). In a Nernstian electrocatalytic reaction, TOF is related to overpotential by the catalytic Tafel equation, Equation (2). TOF is the turnover frequency at the applied potential, *F* is Faraday's constant, *R* is the ideal gas constant, *T* is the temperature, *E*_1/2_ is the catalyst standard reduction potential, and η_app_ is the difference between $E_{\text{CO2/CO}}^{0}$ and the applied potential. It is worth noting, however, that the limitations of the molecular catalytic reactions, including diffusion and side phenomena, can cause ‘peaks’ in catalytic CV waves and truncate the region where the Tafel relationship results in increased activity as the applied potential becomes more negative.

(2) $$\begin{array}{r} \text{TOF}=\frac{\text{TO}{\text{F}}_{\max}}{1+\exp\left[\frac{F}{RT}\left(E_{\text{CO2/CO}}^{0}-E_{1/2}-\eta_{\text{app}}\right)\right]} \end{array}$$

**Figure 3:**
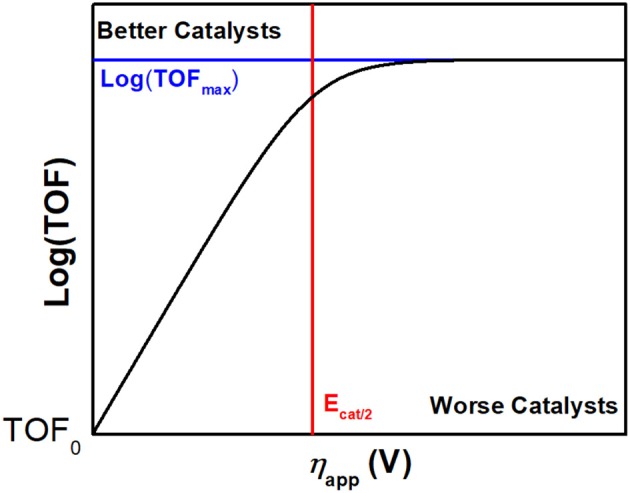
Example of a catalytic Tafel plot of a generic molecular species with regions where better and worse catalysts are located explicitly labeled, along with the position of important benchmarking parameters.

**Figure 4:**
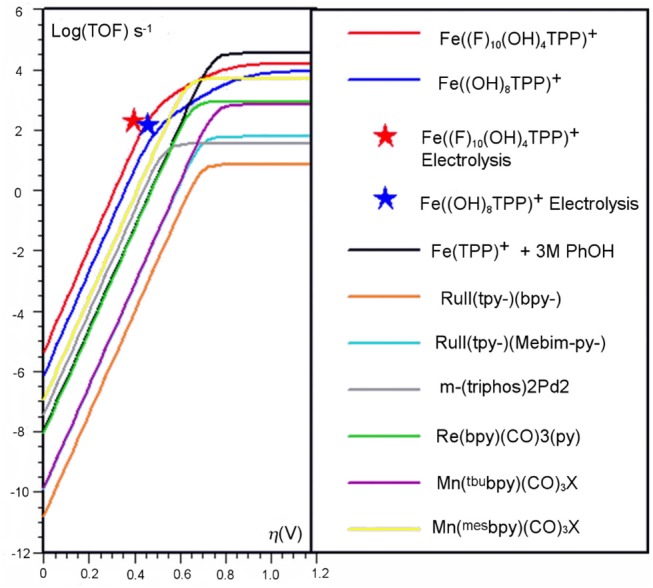
Catalytic Tafel plot showing enhancements from the inclusion of a secondary coordination sphere in the [Fe(TPP)]^+^ system (Costentin et al., 2014b).

To benchmark the kinetic and thermodynamic parameters of different electrocatalysts, Equation (2) can be used to generate catalytic Tafel plots (*nota bene*, these explicitly include the limitations imposed on molecular Tafel behavior mentioned above and enable comparison across different experimental conditions). Figure 3 shows an example of a catalytic Tafel plot for a generic molecular catalyst: “better” catalysts are located at the top left of catalytic Tafel plots, where overpotential is low and TOF_max_ is large and “worse” catalysts are located at the bottom right of a catalytic Tafel plot where the trends in overpotential and TOF_max_ are reversed. Figure 4 shows a Tafel plot comparing reported catalysts.

Finally, simple plots of log(TOF_max_) vs. *E*_cat/2_ can be used to compare catalysts in the same family to look for secondary-sphere effects (Pegis et al., 2017; Costentin and Savéant, 2018b; Nichols et al., 2018b). If there is no secondary-sphere effect, then a linear scaling relationship based on electrochemical driving force should occur; however, if a secondary-sphere effect is present, a deviation from the linearity achieved by comparing inductive substitution effects can be observed. One should utilize caution using this method for comparing catalysts, however, as changes to the reaction mechanism that alter cosubstrate identity, concentration dependence, solvent, and cosolvent effects can greatly impact the catalytic activity of a series of complexes through scaling relationships that are unrelated to the secondary-sphere interactions of interest. Likewise, the effects of Nernstian changes in the experimentally observed potential based on changes in the reaction equilibrium, *K*_eq_, can obscure details if not properly accounted for (Costentin and Savéant, 2018b).

### Secondary-Sphere Effects in [Fe(TPP)]^+^

For [Fe(TPP)]^+^ complexes, increased activity for the electrocatalytic reduction of CO_2_ to CO is observed with the addition of both Lewis and Brønsted acids (Bhugun et al., 1994, 1996; Gennaro et al., 1996). As a result, [Fe(TPP)]^+^ complexes bearing secondary-sphere functionalities exploiting classical Brønsted acid/base push–pull reactions have been the subject of extensive studies (Costentin et al., 2012a, 2014a,b; Sinha and Warren, 2018). In particular, iron 5, 10, 15, 20-*tetrakis*-(2,6-dihydroxyphenyl)-porphyrin ([Fe((OH)_8_TPP)]^+^) was identified as a promising candidate for electrochemical CO_2_ reduction because it positioned proton donors oriented toward the active site in close enough proximity to interact with bound substrate. Experimentally, the pendent –OH moieties were observed to cause a large catalytic current enhancement and a decrease in overpotential (Costentin et al., 2012a). Control experiments using [Fe((OMe)_8_TPP)]^+^, where the –OH groups were converted to –OMe ether moieties, also showed an increased TOF_max_ relative to [Fe(TPP)]^+^; however, the overpotential was much larger than both [Fe(TPP)]^+^ and [Fe((OH)_8_TPP)]^+^. These differences relate to the mechanism of CO_2_ reduction by [Fe(TPP)]^+^ and how key steps are affected by the presence of the pendent –OH moieties (Bonin et al., 2017).

The proposed mechanism for the reduction of CO_2_ by [Fe(TPP)]^+^ requires the electrochemical generation of [Fe(0)TPP]^2−^ at the electrode surface, followed by CO_2_ binding and activation to generate [Fe(I)(TPP)(*η*^1^-${\text{CO}}_{2}^{•-}$)]^2−^. The sacrificial proton donor, AH, stabilizes the binding of the ${\text{CO}}_{2}^{•-}$ radical anion through hydrogen bonding. Concerted protonation and electron transfer from the metal center cause C–O bond cleavage, leading to the formation of [Fe(II)(TPP)(CO)]^0^. Release of CO is facilitated by a comproportionation reaction with a second equivalent of [Fe(0)TPP]^2−^ to generate two equivalents of [Fe(I)TPP]^−^ and one equivalent of CO. We note that a second mechanism has been proposed for Fe(TPP), wherein upon CO_2_ binding, the metal center is oxidized by 2e^−^ from Fe(0) to Fe(II) in the η^1^-CO_2_ adduct before interaction with the proton donor, rather than generating an Fe(I) species (Fukuzumi et al., 2018).

The introduction of a pendent proton source minimally alters the mechanism for CO_2_ reduction but causes catalytic rate increases by favoring several steps of the reaction. Upon the generation of [Fe(0)((OH)_8_TPP)]^2−^, CO_2_ also binds in η^1^ fashion and is activated to the ${\text{CO}}_{2}^{•-}$ radical anion, but stabilization occurs through hydrogen-bonding interactions with the pendent proton donors (Costentin et al., 2012a). Experimentally, this is observed as a prewave to the catalytic feature in CV experiments, which can be more easily examined through additional modulation of electron density at the metal center with the related partially fluorinated derivative [Fe((F)_10_(OH)_8_TPP)]^+^ (Costentin et al., 2014a,b). Subsequent intramolecular protonation balanced by the exogenous proton source can occur, generating η^1^-^•^CO_2_H at an Fe(I) center (Costentin et al., 2014a). The cleavage of the C–OH bond is induced upon further reduction and a concerted intramolecular protonation reaction (again balanced by proton transfer from the sacrificial donor) to regenerate the resting state, Fe(I), of the electrocatalytic cycle and CO product. The enhanced catalytic activity at more negative potentials of the ether-containing control complex may also be explained through an enhancement of this mechanism (the ether groups function as a Brønsted base to orient and enhance the proton activity of the exogeneous proton donor), but this has a relatively lower enhancement effect on the CO_2_ reduction reaction. A comparison of the catalytic activity increases from these effects can be seen through the catalytic Tafel analysis shown in Figure 4.

**Figure 5:**
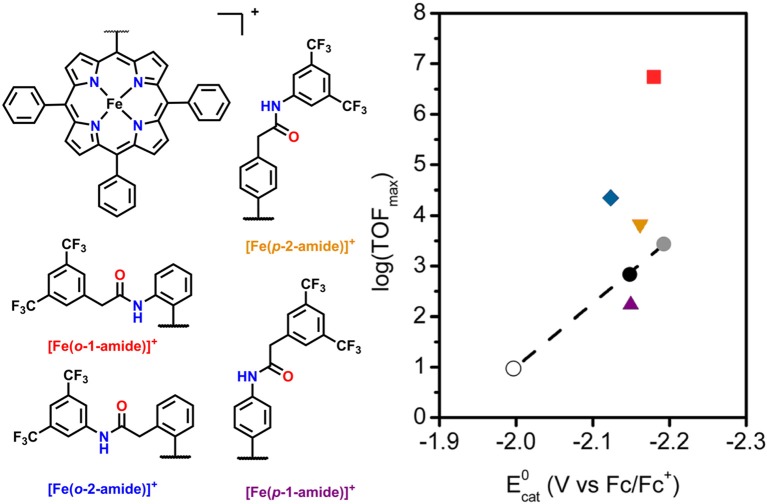
Plot of log(TOF_max_) vs. ${\text{E}}_{\text{cat}}^{0}$ for mono-functionalized porphyrins used to study positional dependence of catalytic enhancement for this system. Circles are [Fe(TPP)]^+^ derivatives which utilize electronic substitution of the phenyl rings for modulation of the redox potential of the Fe^I/0^ couple. The upward purple triangle is [Fe(*p*-1-amide-TPP)]^+^, the downward orange triangle is [Fe(*p*-2-amide-TPP)]^+^, the blue diamond is [Fe(*o*-1–amide-TPP)]^+^, and the red square is [Fe(*o*-2-amide-TPP)]^+^. Reprinted with permission from Nichols et al. (2018b)—published by the Royal Society of Chemistry.

Another area of study for secondary-sphere effects focuses on mono-functionalized porphyrins, which also orient functional groups toward the metal active site. Both the positioning of these functional groups relative to the active site and the p*K*_a_ of the pendent proton source are of importance to the electrocatalytic activity for CO_2_ reduction. In a study by Chang and co-workers the positional effects of pendent amide groups were investigated. It was found that orientation toward the active site and positioning above the active site were both important for catalytic enhancement, as these stabilized catalytic intermediates through hydrogen bonding and facilitated efficient proton transfer (Nichols et al., 2018b). This was quantified experimentally through equilibrium binding constants for CO_2_ determined by rapid-scan CV techniques. When a pendent amide group was attached to the *meso*-phenyl of the porphyrin in the ortho position, [(Fe(*o*-2-amide-TPP)]^+^, the highest catalytic activity was observed. In comparison with the other reported derivatives, a “Goldilocks” relationship was observed, where positioning the amide group either closer or farther did not result in comparable catalytic current enhancement (Figure 5).

**Figure 6:**
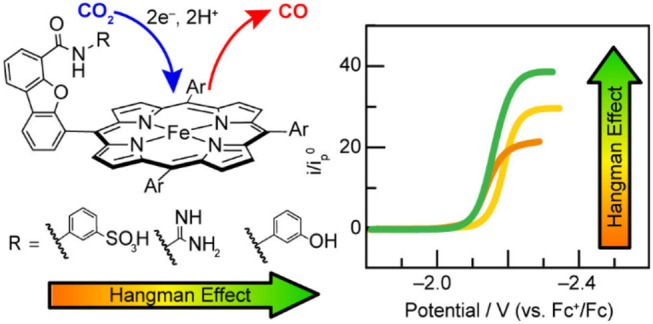
Figure showing effect of p*K*_a_ of the hanging group on CO_2_ reduction activity in hangman [Fe(TPP)]^+^. Reprinted with permission from Margarit et al. (2018). Copyright 2018 American Chemical Society.

Nocera and co-workers found the p*K*_a_ of the pendent proton source was important for the stabilization of CO_2_ binding in the active site of related “hangman-type” porphyrin architectures (Figure 6) (Margarit et al., 2018). Pendent phenol- and guanidinium-based hangman functional groups were predicted by DFT to cause a 2.1–6.6 kcal/mol stabilization of bound CO_2_ within the hangman pocket. Conversely, a sulfonic acid derivative was found to function as a proton donor under the experimental conditions in the absence of applied potential, which was attributed to an estimated p*K*_a_ of ~3 under experimental conditions. The resultant anionic sulfonate derivative showed diminished catalytic activity relative to the porphyrins functionalized with phenol and guanidinium. The anionic charge of the conjugate base, combined with the overall steric bulk of the sulfonate, was proposed to prevent CO_2_ binding within the hangman cleft and contribute minimally to the stabilization of the CO_2_ adduct (Margarit et al., 2018). The pendent phenol-based “hangman-type” architecture was predicted to have the greatest stabilization of CO_2_ binding by DFT calculations and was observed to have the fastest catalytic rate constant by CV methods.

Others have examined the relationship between electrocatalytic activity and the H-bonding ability of pendent residues on triazole-based picket-fence-type [Fe(TPP)]^+^ derivatives with pendent amide functional groups (Sen et al., 2019). In this report, a correlation between log(TOF_max_) and the p*K*_a_ of pendent proton donors was established for a picket fence amide with an encapsulated water molecule in comparison to previously reported [Fe(TPP)]^+^ derivatives. The pendent groups are proposed to contribute to the stabilization of the Fe-bound CO_2_ molecule through hydrogen-bonding interactions and facilitate proton transfer to mediate the rate-determining C–O bond cleavage step.

The studies discussed above have shown that pendent protons can be beneficial for [Fe(TPP)]^+^-based electrocatalysts, but also that careful consideration of steric constraints and the p*K*_a_ of the chosen pendent proton donor under experimental conditions is necessary. Installation of pendent proton groups in porphyrin ligands can have a detrimental effect if their p*K*_a_ and spatial orientation are not chosen carefully (e.g., sulfonic acid, *vide supra*). A recent study has also shown that the choice of solvent can largely alter the catalytic response of [Fe(TPP)]^+^ with an asymmetrically substituted pendent proton moiety (Sinha and Warren, 2018). Using a porphyrin containing a single pendent hydroxy functionality in the 2-position of a single *meso*-phenyl ring, it was demonstrated that the electrocatalytic activity of this system can be greatly hindered by utilizing solvents with strong hydrogen-bonding properties through a comparison of activity in MeCN, DMF, and DMSO. Strong hydrogen bond acceptor solvents like DMF and DMSO lead to a decrease in activity of the catalyst, while in MeCN, the activity approached that reported for [Fe((*ortho*-TMA)TPP)]^5+^, which is the fastest molecular electrocatalyst for CO_2_ reduction reported to date. The authors postulated that the interference of competitive hydrogen bonding between solvent molecules leads to slowed electrochemical kinetic parameters (Sinha and Warren, 2018). This suggests that more rigorous interrogations of functional group cooperativity (specifically the number and type of functional groups) and the interactions of functional groups with solvent and proton donors will offer additional insight into these mechanisms. Indeed, Costentin and Savéant described the origin of catalytic enhancement by pendent proton relays (Costentin and Savéant, 2018b). Boosting of electrocatalytic activity by pendent proton donors occurs when the forward rate constant of proton transfer from the pendent group to the active site is competitive with that of exogeneous proton donor directly to the active site (under the assumption that reprotonation of the pendent donor is extremely rapid). Should these forward rate constants not be well-matched, inefficiency in the proton relay mechanism will limit the ultimate catalytic current enhancement.

Further studies on this class of catalysts have modulated the potential of the Fe^I/0^ reduction where the catalytic response occurs by examining electron-withdrawing perfluorophenyl-substituted tetraphenylporphyrins ([Fe(F_5_TPP)]^+^, [Fe(F_10_TPP)]^+^, and [Fe(F_20_TPP)]^+^, where F_5_ corresponds to a single perfluorinated phenyl ring, etc.) (Azcarate et al., 2016). This is a purely electronic effect, perturbing the continuum of electronic distribution in the ligand–metal manifold. The inclusion of proximal ionic charges has also been explored with trimethylanilinium- ([Fe(-*ortho*-TMA-TPP)]^5+^ and [Fe(-*para-*TMA-TPP)]^5+^) and sulfonato-functionalized porphyrins ([Fe(-*para-*sulfonato-TPP)]^3−^) to understand the role of through-space electrostatic interactions (Azcarate et al., 2016). The perfluorinated derivatives demonstrated a relationship between the catalytic response and the Fe^I/0^ potential: as the potential of that redox couple becomes more positive, log(TOF_max_) decreases in a linear fashion, corresponding to a relative decrease in added electron density at the metal center upon electrochemical reduction (Azcarate et al., 2016; Costentin et al., 2018). However, for [Fe(TPP)]^+^ substituted with a charged functional group (*para*–${\text{SO}}_{3}^{2-}$, *para*–${\text{NMe}}_{3}^{+}$, and *ortho*–${\text{NMe}}_{3}^{+}$), log(TOF_max_) increases linearly as the redox potential of the Fe^I/0^ couple shifts to more positive potentials. [Fe-*ortho*-TMA-TPP]^5+^ has the most positive Fe^I/0^ redox potential of any [Fe(TPP)]^+^-based electrocatalyst reported to date, as well as largest log(TOF_max_) value. To understand this, one can once again imagine the CO_2_ binding mode wherein a single electron generates radical ${\text{CO}}_{2}^{•-}$ anion, which is stabilized by the charged groups close to the active site. It is compelling that such dramatic enhancements should be observed, but this could suggest that the reaction pathway might also be significantly altered from other [Fe(TPP)]^+^ derivatives, as the effect is an inverse scaling relationship to that predicted for purely inductive reasons (Azcarate et al., 2016).

The data points from charge-based functionalization are relatively limited; the proposal of design principles for new systems for molecular CO_2_ reduction systems requires additional information on these effects. For instance, the synthesis of further positional isomers of charged systems could give more evidence that a scaling relationship exists based on the distance between the charged moiety and the active site. Ultimately, it is clear that the inclusion of charge should seek to explore deliberate manipulation of the known mechanism to achieve the greatest enhancement effect.

### Re(bpy)(CO)_3_X

**Figure 7:**
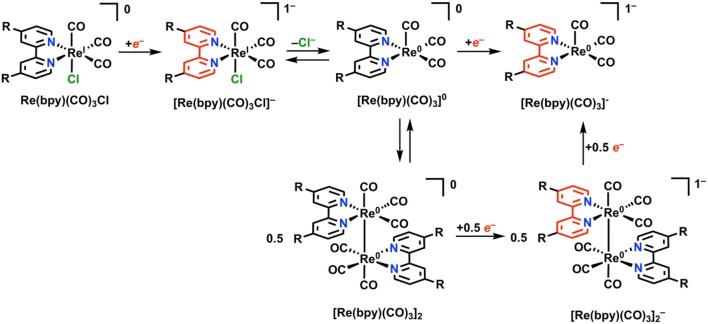
Faradaic reduction of Re(bpy)(CO)_3_X. Adapted with permission from Machan et al. (2014b). Copyright 2014 American Chemical Society.

Re(bpy)(CO)_3_X (where bpy is a 2,2′-bipyridine, often additionally functionalized in a symmetric fashion, and X is a halide anion or solvent molecule) is active for the electrocatalytic reduction of CO_2_ to CO in near quantitative fashion (Hawecker et al., 1984; Grice and Kubiak, 2013). Under Faradaic conditions, this complex is proposed to undergo reduction according to Figure 7. Initial reduction is localized at the bpy ligand, followed by loss of Cl^−^ from [Re(bpy)(CO)_3_Cl]^−^ *via* an overall EC mechanism to generate the neutral five-coordinate species [Re(bpy)(CO)_3_]^0^. At this stage, two separate mechanisms for reduction can occur: 1) a single reduction assigned to the ligand framework can occur, generating the catalytically active monoanionic species [Re(bpy)(CO)_3_]^−^. Commonly, the [Re(bpy)(CO)_3_Cl]^−^ species is stable long enough on the CV timescale that [Re(bpy)(CO)_3_]^−^ forms instead at the second reduction with Cl^−^ loss. 2) Following initial single-electron reduction and Cl^−^ loss, a Re–Re bond between two equivalents of [Re(bpy)(CO)_3_]^0^ can form to generate Re(bpy)(CO)_3_]_2_. Dimer formation requires two sequential reductions to cleave the Re–Re bond and form the [Re(bpy)(CO)_3_]^−^ active species. The formation of the metal–metal bond is slow under most conditions because of the persistence of [Re(bpy)(CO)_3_Cl]^−^ on the CV timescale and is most often outcompeted by the unimolecular pathway to [Re(bpy)(CO)_3_]^−^ (Grills et al., 2014).

There are also two possible mechanisms for CO_2_ reduction under electrocatalytic conditions. First, a relatively slower bimolecular process involving two equivalents of [Re(bpy)(CO)_3_]^0^ can occur, resulting in a net reductive disproportionation of two equivalents of CO_2_ into one each of CO and ${\text{CO}}_{3}^{2-}$ (Sullivan et al., 1985). Second, a unimolecular catalytic process with [Re(bpy)(CO)_3_]^−^ as the resting state, where the two-electron reduction of CO_2_ occurs at a single Re center (Sullivan et al., 1985; Keith et al., 2013). As is observed with the [Fe(TPP)]^+^-based electrocatalysts above, there are observable enhancements in TOF_max_ and catalyst stability for CO_2_ reduction upon the addition of a sacrificial proton donor (Wong et al., 1998).

**Figure 8:**
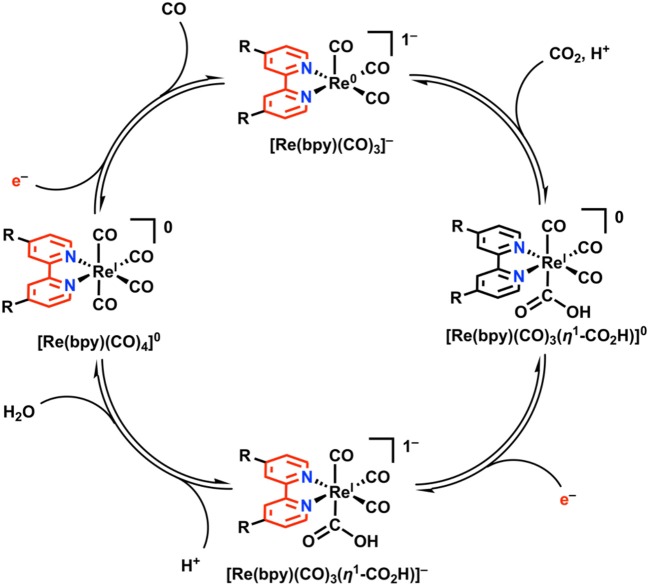
2e^−^/2H^+^-dependent catalytic cycle for Re(bpy)(CO)_3_X in the reduction of CO_2_ to CO. Adapted with permission from Machan et al. (2014b). Copyright 2014 American Chemical Society.

In the proposed mechanism for electrocatalytic CO_2_ reduction, a monoanionic five-coordinate species [Re(bpy)(CO)_3_]^−^ is invoked as the active species which binds CO_2_ (Figure 8) (Keith et al., 2013). This has been validated experimentally through direct synthesis of [Re(bpy)(CO)_3_]^−^ using chemical-reducing agents, with subsequent spectrochemical studies demonstrating a kinetic preference for CO_2_ over H^+^ (Smieja et al., 2012; Sampson et al., 2013). Re(bpy)(CO)_3_(η^1^-CO_2_H) is initially formed upon the binding and activation of CO_2_ by [Re(bpy)(CO)_3_]^−^ with a proton donor present. Further reduction generates an anionic species, [Re(bpy)(CO)_3_(η^1^-CO_2_H)]^−^, at which point C–O bond cleavage is facilitated by an exogenous proton source to generate water and the neutral species, [Re(bpy)(CO)_4_]^0^. CO release from the 19e^−^ complex [Re(bpy)(CO)_4_]^0^ is facile, and additional reduction regenerates the resting [Re(bpy)(CO)_3_]^−^ state. In the Re(bpy)(CO)_3_X catalyst family, most work has focused on modulating the steric and electronic properties of the bipyridine ligand in attempts to modulate the reducing power and activity of Re. Generally, the use of electron-rich bpy ligands like 4,4′-di-*tert*-butylbpy enhances the activity of the catalyst by creating a more nucleophilic Re center upon reduction, albeit at larger overpotentials (Clark et al., 2018).

Kubiak and co-workers have demonstrated, however, that steric considerations can supersede electronic effects in this class of catalysts. Re(bpy)(CO)_3_X-type complexes were synthesized with 3,3′- and 5,5′-dimethyl bpy (Chabolla et al., 2014). The methyl groups in those ring positions have identical Hammett parameters, so for purely electronic reasons, their respective electrocatalytic activities with a [Re(CO)_3_]^+^ core would be expected to be similar. When compared to the unsubstituted parent compound, however, the 5,5′-dimethyl bpy complex shows increased catalytic current at slightly more negative potentials, while the 3,3′-dimethyl bpy shows decreased catalytic current at nearly the same potential. This is rationalized through the steric hindrance from the methyl groups at the 3,3′-positions, which is observed in the non-planarity of the bpy ligand in the crystal structure of the precatalyst. Previous experimental and computational studies have postulated that a key mechanistic component is the participation of π^*^ orbitals in the bpy ligand in the redox mechanism, suggesting that an inability to achieve a higher degree of planarity upon reduction diminishes catalytic activity for 3,3′-dimethyl bpy-based Re catalysts (Smieja et al., 2012; Benson et al., 2013; Keith et al., 2013; Chabolla et al., 2014). Since the distribution of added electron density between the bpy ligand and the Re metal center has been identified as important to the selectivity for CO_2_ over H^+^ as the electrophile of choice; it is noteworthy that an steric perturbation of the HOMO of the active species should have this effect.

The effect of secondary-sphere modifications on the Re(bpy)CO_3_X system was explored through the incorporation of –OH groups at the 4,4′- and 6,6′-positions of the bpy, with the goal of using them both as a pendent proton source and to contribute electron density to the bpy ligand (Manbeck et al., 2015). In this case, the authors found atypical behavior during electrocatalysis: the O–H bonds of the bipyridine ligand are cleaved by stepwise electrochemical reduction, which they propose leads to dearomatization of the doubly reduced bpy ligand. By isolating the deprotonated species in a chemical fashion, the authors were able to give both UV-vis and IR spectroscopic evidence—which aligned well with infrared spectroelectrochemistry (IR-SEC), UV-vis spectroelectrochemistry (UV-vis-SEC), and DFT calculations—supporting this hypothesis. Interestingly, only the 4,4′-dihydroxybpy complex is active as an electrocatalyst for CO_2_ reduction, while the 6,6′-dihydroxybpy complex completes only ~1 TON for CO, despite having nearly identical electrochemical properties under Faradaic conditions by CV. This result suggests slow CO release and decomposition of intermediates at applied potentials limit activity for the 6,6′-dihydroxybpy derivative.

Further work with monomeric Re(bpy)(CO)_3_X electrocatalysts showed promising results with the incorporation of peptide linkages of varying lengths containing proton relays and hydrogen-bonding groups on the bpy backbone (Chabolla et al., 2017). Through electrochemical experiments, 2D NMR spectroscopy, and molecular dynamics modeling, the study showed that an optimal chain length of five peptides allows for the peptide backbone to adopt conformations which allow for intramolecular interactions on the NMR timescale. Importantly, this study showed the Re(bpy)(CO)_3_X system to be stable under peptide synthesis conditions, allowing for insertion of the complex into peptide linkages at any desired point (Chabolla et al., 2017).

The asymmetric incorporation of a thiourea tether into Re(bpy)(CO)_3_X systems has been shown to be a successful strategy for enhancing the CO_2_ reduction activity (Haviv et al., 2018). Interestingly, the sulfur atom of the thiourea tether was shown to bind CO_2_ prior to reduction of the Re catalyst, which is expected to lower the reorganization energy penalty expected for the reduction of the linear CO_2_ molecule. This interaction was shown to work synergistically with the reduced Re state, as the thiourea moiety also enhanced the catalytic response by acting as a pendent proton donor capable of beneficial hydrogen-bonding interactions and facilitating C–O bond cleavage. Added Brønsted acids inhibited catalytic activity, likely the result of competitive interactions with the pendent thiourea tether. In a separate study on the asymmetric incorporation of phenolic pendent proton sources to Re(bpy)(CO)_3_X, catalysis was ‘turned on’ at lower overpotentials, specifically the first reduction potential by CV in the two complexes studied (Rotundo et al., 2019). These complexes did, however, suffer from low Faradaic efficiencies for CO when no external proton source was present. Each of these catalysts also had issues with electrode absorption phenomena, a deleterious reaction with inhibitory consequences for nominally molecular electrocatalysts.

A Re(bpy)-based CO_2_ reduction catalyst which features an imidazolium group as a charged residue in the secondary sphere reported by Nippe and co-workers was observed to cause changes in redox properties and mechanism compared to unfunctionalized Re(bpy)(CO)_3_X (Sung et al., 2017). It was proposed that the C_2_-H carbon of the imidazolium moiety was important for the catalytic enhancement through an alteration of mechanism: theoretical methods suggested that hydrogen bond-like or electrostatic C_2_-H—X (X = Cl^−^, ${\text{CO}}_{2}^{-}$, or H_2_O) interactions change the ground state energies of intermediates relevant to the catalytic cycle. These assignments were supported experimentally through testing of a control complex where the imidazolium C_2_-H was replaced by C_2_-CH_3_, and the non-linear dependence of the catalytic activity of each complex on [H_2_O], which is anomalous to the archetypal electrocatalytic response of Re(bpy)(CO)_3_X. It was postulated that a reduction-first mechanism for CO_2_ reduction was occurring, where reduction of the Re(I)(η^1^-CO_2_H) adduct preceded protonation and C–O bond cleavage, as is classically seen in the unfunctionalized complex (Figure 8).

The effects of charge on the electrocatalytic activity of Re(bpy)(CO)_3_X were studied in a series of polymeric frameworks using a series of charged monomers (Sahu et al., 2017). Three norbornenyl-based polymers containing either positively charged quaternary ammonium, neutral phenyl, or negatively charged trifluoroborate moieties were generated through ROMP and covalently end-labeled with a Re(bpy)(CO)_3_X-based terminating reagent. Electrochemical studies in acetonitrile indicated that the polymers containing quaternary ammonium salts exhibited catalytic behavior at a significantly more positive potential (~300 mV) than the neutral polymer, which behaved consistently with unfunctionalized Re(bpy)(CO)_3_X. The incorporation of negatively charged groups caused a shift to more reducing potentials, and catalytic activity was not observed in the solvent window. The incorporation of known catalysts onto a polymeric framework with the ability to tune reduction potential is a possible precursor to highly ordered structures such as thin films, abiotic metalloproteins, porous catalytic membranes, and cationic nanoparticles for use in devices.

### Mn(bpy)(CO)_3_X

**Figure 9:**
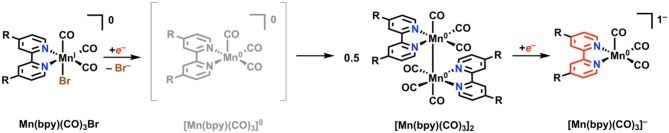
Faradaic reduction mechanism of Mn(bpy)(CO)_3_X. Adapted with permission from Machan et al. (2014b). Copyright 2014 American Chemical Society.

Unpublished results referenced by Johnson et al. in 1996 stated that Mn(bpy)(CO)_3_X, unlike its third-row congener Re, was inactive for electrocatalytic CO_2_ reduction under aprotic conditions (Johnson et al., 1996). A more recent examination by Deronzier and co-workers in 2011 repeated this result, but also showed that the addition of a weak proton donor facilitated a significant and selective electrocatalytic response for CO_2_ reduction to CO (Bourrez et al., 2011). One important mechanistic detail about the Mn(bpy)(CO)_3_X system is that upon reduction by a single electron and subsequent loss of X, a Mn–Mn dimer [Mn(bpy)(CO)_3_]_2_ can rapidly form with rates approaching the diffusion limit (Figure 9) (Grills et al., 2014). The formation of this dimer has two detrimental effects: (1) it increases the electrochemical driving force required to generate the catalytically active monoanionic five-coordinate [Mn(bpy)(CO)_3_]^−^ species required for CO_2_ reduction (the Mn–Mn bond requires more reducing potentials to cleave), and (2) it reduces the activity of the complex toward CO_2_ reduction as a non-catalytic competing pathway (Grice and Kubiak, 2013; Smieja et al., 2013).

**Figure 10:**
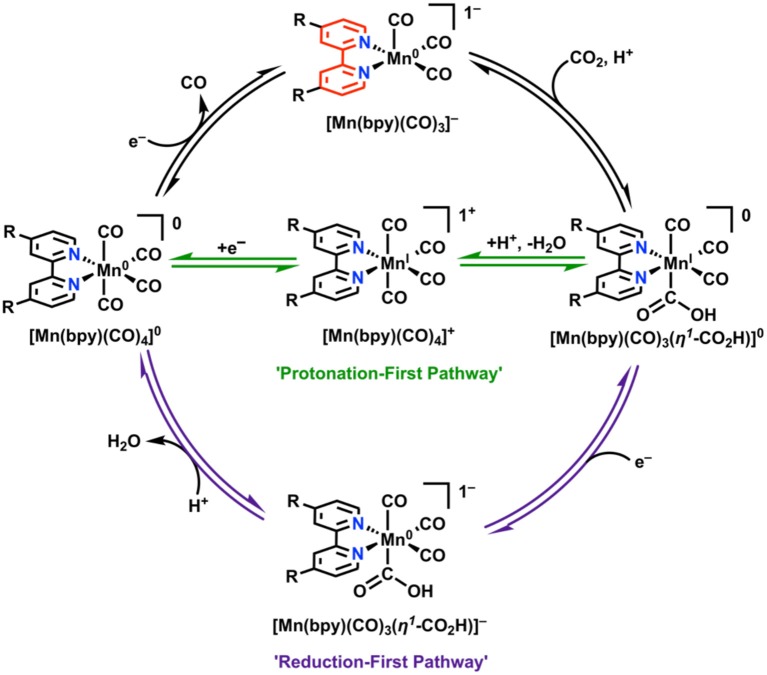
“Protonation-first” and “reduction-first” pathways for CO_2_ reduction by Mn(bpy)(CO)_3_X (Riplinger et al., 2014).

In attempts to combat these detrimental effects from dimerization, a bulky bpy analog was designed (6,6′-dimesitylbpy) (Sampson et al., 2014; Sampson and Kubiak, 2016). This ligand framework proved to be effective in eliminating the dimerization reaction. Rather than two irreversible 1e^−^ waves on the reductive sweep as seen in Mn(bpy)(CO)_3_X, a single reversible 2e^−^ wave was observed (Sampson et al., 2014). This leads to the formation of monomeric, anionic [Mn(mesbpy)(CO)_3_]^−^ at 300 mV more positive potentials than in the case of the original Mn(bpy)(CO)_3_X complexes (Sampson et al., 2014). In the presence of CO_2_ and a proton source, the 2e^−^ reversible feature becomes irreversible and shifts toward positive potentials, which is indicative of CO_2_ binding (Sampson et al., 2014). This mechanistic difference was confirmed through control experiments, IR-SEC (Ashley and Pons, 1988; Zavarine and Kubiak, 2001; Best et al., 2008; Kaim and Fiedler, 2009; Machan et al., 2014b), and the direct synthesis of the active species. A significant catalytic response does not occur in the presence of Brønsted acids until potentials are similar to those that are catalytic for the unfunctionalized parent complex (Sampson et al., 2014). Rapid C–O bond cleavage in the hydroxycarbonyl intermediate [Mn(mesbpy)(CO)_3_(η^1^-CO_2_H)] does not occur until “overreduction” to generate [Mn(mesbpy)(CO)_3_(η^1^-CO_2_H)]^−^ at potentials 400 mV more negative than the 2e^−^ reversible feature (Figure 10) (Riplinger et al., 2014). To take advantage of the initial CO_2_ binding event by [Mn(mesbpy)(CO)_3_]^−^ at more positive potentials, a subsequent report used Lewis acid additives (Sampson and Kubiak, 2016). This strategy proved successful, as the addition of Mg^2+^ ions as cosubstrate to solution aided in C–O bond cleavage at the potential where CO_2_ binding occurs, greatly reducing the overpotential required for the generation of CO from CO_2_ (Sampson and Kubiak, 2016).

Initial attempts to incorporate pendent proton relays into the Mn(bpy)(CO)_3_X framework involved asymmetric attachment of phenol moieties onto the bpy ligand (Franco et al., 2014; Agarwal et al., 2015). This led to enhanced activity in comparison to the parent Mn(bpy)(CO)_3_X complex, including activity in the absence of a proton source; however, the competitive dimerization reaction was still apparent in each case (Franco et al., 2014; Agarwal et al., 2015). Converting the pendent –OH groups to ethers with methoxy groups showed insignificant or no catalytic activity in control studies for these derivatives.

To extend these studies to symmetrically functionalized systems, Rochford and co-workers synthesized a methoxy ether-containing analog of the bulky bpy ligand, 6,6′-(2,6-dimethoxyphenyl)bpy [(MeO)_2_Ph]_2_bpy (Ngo et al., 2017). A combined experimental and theoretical analysis showed that through the introduction of sufficiently acidic exogeneous proton sources, the slower “protonation-first” pathway seen in the original bulky bpy study could be “turned on” at lower overpotentials in comparison to the “reduction-first” pathway at more negative potentials (Ngo et al., 2017). In the protonation-first pathway, a bound hydroxycarbonyl is formed, and further protonation by a sufficiently strong acid can lead to the facilitation of C–O bond cleavage prior to reduction and release of CO at more positive potentials than the reduction-first pathway (Figure 10). In the reduction-first pathway, a bound hydroxycarbonyl is again formed; however, without a sufficiently strong acid present, reduction of the hydroxycarbonyl must occur at more negative potentials prior to protonation and C–O bond cleavage.

**Figure 11:**
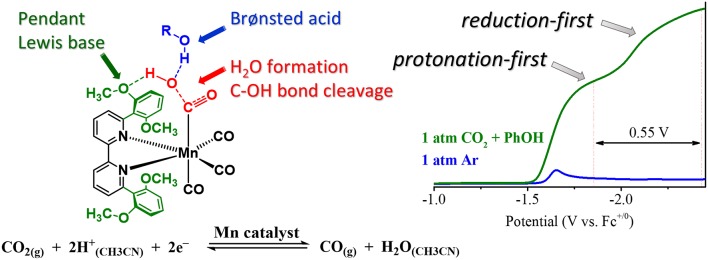
Figure showing proposed transition state which occurs to turn on a low-overpotential, secondary-sphere-promoted mechanism for CO_2_ reduction with Mn((OMe)_2_bpy)(CO)_3_X. Adapted with permission from Ngo et al. (2017). Copyright American Chemical Society 2017.

The acid dependence of the “protonation-first” and “reduction-first” pathways for Mn([(MeO)_2_Ph]_2_bpy)(CO)_3_X was rationalized through a hydrogen-bonding interaction between the Mn-bound η^1^-CO_2_H hydroxycarbonyl species and the pendent Lewis base groups located on the ligand (Figure 11). This hydrogen bond donor–acceptor interaction was also proposed to facilitate the subsequent transfer of a second equivalent of a sufficiently strong Brønsted acid additive to protonate and facilitate C–O bond cleavage, ultimately producing H_2_O and Mn-bound CO (Ngo et al., 2017). In the presence of a proton source of insufficient acidity, the reduction of the bound η^1^-CO_2_H hydroxycarbonyl species is instead required before protonation and C–O bond cleavage can occur, as is observed with the “overreduction” of the Mn catalyst with the “bulky” bpy ligand (Ngo et al., 2017). Importantly, no metal–metal dimerization reaction was observed for this ligand.

Recently, a charged imidazolium functionality was also introduced asymmetrically into the Mn(bpy)(CO)_3_X system (Sung et al., 2019). A series of derivatives examining hydrogen-bonding effects showed activity for CO_2_ reduction at potentials of only −1.4 V vs. Fc^+^/Fc in the presence of water. This was postulated to originate from a synergistic effect between the pendent imidazolium functionality and a network of water molecules in the solvation shell that facilitated CO_2_ reduction. The combined use of charge and hydrogen-bonding donors shows promise for lowering the catalytic overpotential, but further work is needed to enhance Faradaic efficiencies to match other catalyst platforms in this family.

### Ni(cyclam)]^2+^

The electrocatalytic activity of a [Ni(cyclam)]^2+^ (cyclam = 1,4,8,11-tetraazacyclotetradecane) derivative was first reported in 1980 by Fisher and Eisenberg (1980). It has been the subject of continuous study because of its tolerance for a wide range of acid strengths and solvent systems without a loss of catalytic activity (Beley et al., 1984; Barefield et al., 1986; Balazs and Anson, 1993; Kelly et al., 1999; Schneider et al., 2012). The activity of Ni(cyclam)]^2+^ for electrocatalytic reduction of CO_2_ to CO in the presence of water as a Brønsted acid source was attributed specifically to the *Trans I* isomer (one of six possible isomers based on the orientation of the H atoms on the four metal-coordinated secondary amines in the macrocycle; all four H atoms are cofacial). This isomer is the most favorable for high CO_2_ reduction activity due to the hydrogen bond donor–acceptor interactions between the ligand NH groups and a Ni-bound CO_2_ molecule (Froehlich and Kubiak, 2012). Furthermore, DFT calculations have indicated that the *trans I* isomer has a more stable CO_2_ adduct than the *trans III* isomer (two H atoms are cofacial) by approximately 3 kcal/mol (Song et al., 2014).

**Figure 12:**
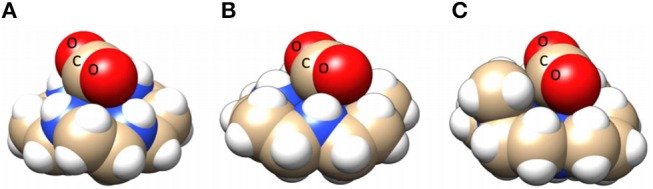
Space-filling models of *trans I* [Ni(cyclam)(CO_2_)]^+^ **(A)**, *trans III* [Ni(cyclam)(CO_2_)]^+^ **(B)**, and [Ni(DMC)(CO_2_)]^+^ **(C)**. Structures indicate that enhanced CO_2_ reduction in the case of *trans I* [Ni(cyclam)(CO_2_)]^+^ may be due to increased stabilization of the CO_2_ adduct through increased hydrogen bond donors oriented toward the hydrogen bond accepting O atoms on the CO_2_ adduct. Used with permission from Song et al. (2014). Copyright American Chemical Society 2014.

Further evidence for the importance of the amine protons and these hydrogen-bonding interactions is found through a comparison with the *N*-alkylated derivatives [Ni(dimethylcyclam)]^2+^ and [Ni(tetramethylcyclam)]^2+^, where greatly diminished activity for electrocatalytic CO_2_ reduction is observed (Froehlich and Kubiak, 2012). The absence of hydrogen bond-induced stabilization effects and increased steric parameters make CO_2_ binding less facile in the *N-*alkylated derivatives (Figure 12). Pendent proton donor effects have been shown with other functional groups as well: the introduction of a carboxylic acid on the carbon backbone of Ni(cyclam)]^2+^ improves its activity by making it stable and selective for CO_2_ reduction down to pH 2 at similar overpotentials to other reported water-soluble CO_2_ reduction catalysts (Neri et al., 2016).

The use of an exogenous urea additive with Ni(cyclam)]^2+^ was shown to improve catalytic activity for CO_2_ reduction for similar reasons (Nichols and Chang, 2018). This study demonstrated that the urea additive acted as a cocatalyst for the system, and it was proposed that its unique structure allows for it to promote CO_2_ reduction through the formation of multipoint hydrogen bonds with the bound CO_2_ adduct [Ni(cyclam)(η^1^-CO_2_)]^+^ (Nichols and Chang, 2018). This conclusion was supported through the introduction of multiple cationic and neutral additives with similar p*K*_a_s. In each case, the cocatalytic response observed with urea was not present (Nichols and Chang, 2018). Although the urea additive was not tethered to cyclam, it is conspicuous that it should have a cocatalytic role with both Ni(cyclam)]^2+^- and Re(bpy)-based catalysts for CO_2_ reduction, *vide supra*.

### Multimetallic Systems

**Figure 13:**
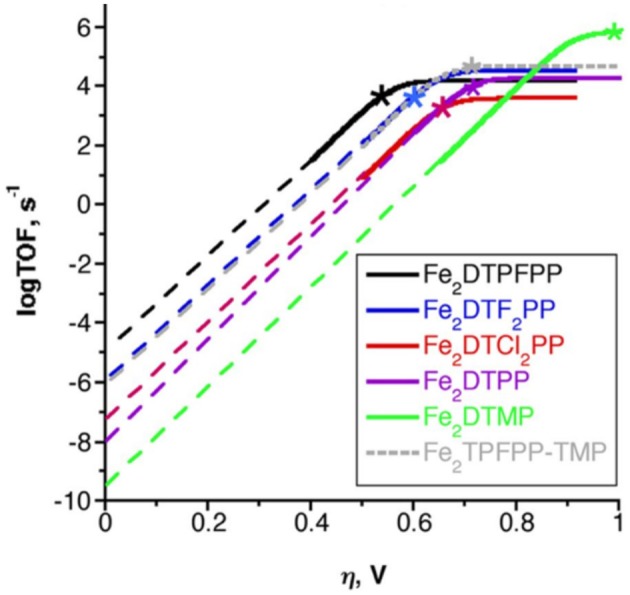
Catalytic Tafel plot showing overpotential tuning in cofacial Fe(TTP) through the introduction of electron with drawing substituents on the phenyl groups of the porphyrin system. Adapted from Zahran et al. (2016).

Multimetallic systems are important examples of secondary-sphere interactions, because they are common to the enzymes which catalyze CO_2_ reduction chemistry (Dobbek et al., 2001b, 2002). In many cases, these multimetallic systems work cooperatively to both store excess charge and to activate the molecule of interest using “push–pull” donor–acceptor effects. Homobimetallic cofacial [Fe(TPP)]^+^ systems connected by a phenylene bridge were synthesized to generate a bimetallic species from the well-known [Fe(TPP)]^+^ system discussed above (Mohamed et al., 2015). By tuning the Fe–Fe distance through synthetic modification, CO_2_ binding could be induced at the Fe^3+/2+^ wave rather than the Fe^1+/0^ as observed in most [Fe(TPP)]^+^ systems (Mohamed et al., 2015; Bonin et al., 2017). This was rationalized through the expected Fe–Fe distance of 3.2–4.0 Å in the *ortho*-bridged system, which would be suitable for binding the linear CO_2_ molecule. In contrast, the *meta*-bridged system was expected to have a significantly shorter separation and showed diminished activity similar to monomeric [Fe(TPP)]^+^ (Mohamed et al., 2015). The *ortho*-bridged system has significant catalytic activity (TOF_max_ = 4300 s^−1^) and high Faradaic efficiency for CO (95%) at an overpotential of ~0.7 V in the presence of 10% H_2_O in DMF, which is a significant improvement over monomeric [Fe(TPP)]^+^. The overpotential for this class of homobimetallic catalyst could be further tuned using electron-withdrawing and -donating substituents on the phenyl rings of each [Fe(TPP)]^+^ unit (Figure 13) (Zahran et al., 2016). When the overpotential was synthetically tuned to ~0.4 V with electron-withdrawing groups, a ~3-fold decrease in activity in comparison to the parent phenyl-functionalized dimer was observed (Zahran et al., 2016). Chang and co-workers have recently demonstrated that these beneficial multimetallic effects extend beyond bimetallic systems using a porous organic cage containing six [Fe(TPP)]^+^ units, which was active for CO_2_ reduction in aqueous solutions (Smith et al., 2018).

A multimetallic Fe carbonyl cluster system with an interstitial main group element [Fe_4_X(CO)_12−n_L_n_]^−^ (X = N or C, L = another ligand, n = 1 or O) has been developed by the Berben group for the reduction of CO_2_ to formate under both aqueous and non-aqueous conditions (Rail and Berben, 2011; Taheri et al., 2015; Taheri and Berben, 2016; Loewen et al., 2017). This cluster is proposed to generate formate *via* an intermediate bridging hydride. The bridging hydride on the cluster motif has the ideal hydricity and p*K*_a_ to selectively generate formate in the presence of CO_2_, preventing the competitive formation of H_2_ (Loewen et al., 2017). Pendent proton groups in this system alter the selectivity of this catalyst family from nearly quantitative generation of formate to the generation of H_2_, which highlights the importance of controlling the kinetics of substrate delivery in determining selectivity for competitive CO_2_ and H^+^ reduction reactions (Loewen et al., 2016).

The development of bimetallic Re complexes based on Re(bpy)(CO)_3_X and its derivatives has been of recent interest for both photocatalytic (Bruckmeier et al., 2012) and electrocatalytic processes due to the concentration-dependent formation of binuclear intermediates as part of the catalytic cycle (Machan et al., 2014a, 2016; Wilting et al., 2017; Yang et al., 2018). In electrocatalytic systems, the first bimolecular Re system studied utilized acetoamidomethyl modified bpy to generate a supramolecular catalyst system *in situ*. This system operated at more positive potentials (~250 mV) in MeCN than the 4,4′-dimethylbpy-based control complex as the result of a hydrogen-bonded dimer active state, albeit with a lower TOF and FE than the unimolecular process (Machan et al., 2014a). To further probe this hydrogen bond-based dimer system, a subsequent report focused on a heterobimetallic Re–Mn construct using a 1:1 mixture of acetoamidomethyl-modified Re(bpy)(CO)_3_X with acetoamidomethyl-modified Mn(bpy)(CO)_3_X (Machan and Kubiak, 2016). Results from this study indicated a cooperative heterobimetallic pathway was operative, and it was proposed that the Mn center was activating CO_2_ followed by protonation to generate a Mn-bound hydroxycarbonyl species within the heterobimetallic dimer. Enhanced reduction-first pathway kinetics were initiated in this case by electron transfer from the reduced Re species present in the dimer.

Further modifications to this supramolecular system replaced the acetoamidomethyl unit with an amide-linked PhOH-containing tyrosine functional group (Machan et al., 2016). This modification led to an increased TOF_max_ in comparison to the initial complex and near quantitative Faradaic efficiency; mechanistic studies where the PhOH unit was substituted for a phenyl ring showed the pendent –OH functionality was essential for improving catalytic activity in the bimetallic mechanism (Machan et al., 2016). The “soft” non-covalent linkages used in this strategy are reminiscent of biological active sites: the catalytic system can adopt a variety of conformations on the potential energy surface facilitated by weaker interactions instead of more rigid systems reliant on distance or conformational tuning through purely synthetic means. The success of this approach is dependent on how well the weak interactions overcome the added diffusional component of the bimolecular reaction mechanism.

Work to generate rigid homobimetallic Re(bpy)(CO)_3_X systems where the metal centers are in close proximity has been achieved using an anthracene linker by Jurss and co-workers (Yang et al., 2018). This complex can be isolated as *cis* or *trans* isomers through chromatography, which alters the relative positioning of the Re centers (Yang et al., 2018). The *cis* isomer, in which the Re centers are in close proximity to one another, outperformed both a monometallic anthracene control complex and the *trans* isomer of the homobimetallic species in catalytic studies. Another covalently linked homobimetallic system with an imidazole–pyridine bridge was examined by Siewert and co-workers (Wilting et al., 2017). The observed catalytic activity of the homobimetallic species outperformed the mononuclear control complex, which was inactive for CO_2_ reduction (Sinha et al., 2017). The introduction of the phenol linker between the two imidazole–pyridine arms to generate a proton relay in close proximity to the active site of the two metal centers was also examined (Wilting et al., 2017). The pendent proton source enhanced the activity of the bimetallic Re complex, with Faradaic efficiencies for CO of ~60% (Wilting et al., 2017).

## Emerging Systems

**Figure 14:**
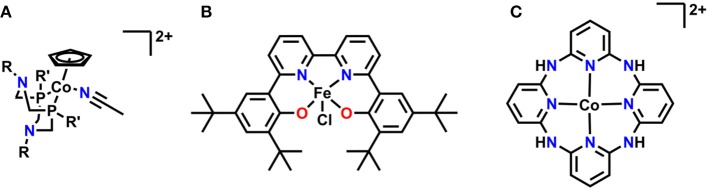
**(A)** Co(Cp)(P_2_N_2_) utilizing pendent tertiary amines for reduction of CO_2_ to formate. **(B)** Fe(^tbu^dhbpy)CI is protonated at the Fe-coordinating O atoms upon reduction and reduces CO_2_ to formate. **(C)** Macrocyclic–aminopyridyl complex which reduces CO_2_ to CO.

Several relatively new systems have also been reported where pendent functional group interactions are essential to the overall mechanism (Figure 14). A recent report on a series of cobalt(cyclopentadienyl)(P_2_N_2_) complexes by Artero and co-workers showed that pendent tertiary amines enabled selective formate generation from CO_2_ (Roy et al., 2017). DFT calculations suggest that a reaction-defining transition state occurs where one of the pendent amines forms a hydrogen-bonding interaction with water to align it with CO_2_ as simultaneous hydride transfer occurs from the Co center, lowering the overall transition state energy and enhancing activity (Roy et al., 2017). Work in our own group has identified a Schiff base-type ligand based on bpy as a promising new direction: the Fe(III) derivative is active for the reduction of CO_2_ to formate with PhOH as a proton source. Mechanistic investigations suggest the Fe-bound oxygen atoms act as a site for protonation upon initial reduction of the complex, generating a pendent proton source for the reaction at applied potential (Nichols et al., 2018a). A macrocyclic, aminopolypyridyl Co complex from Marinescu and co-workers generated CO in a near quantitative fashion in the presence of MeOH as a proton source (Chapovetsky et al., 2016, 2018). Upon alkylation of the pendent amine functionalities, a two-fold decrease in activity occurred, suggesting that the pendent protons on the amines linking the pyridyl groups play an important role as hydrogen bond donors during CO_2_ reduction (Chapovetsky et al., 2016, 2018). The activity and selectivity of all these platforms are promising for future studies on optimizing secondary-sphere effects.

## Conclusions and Outlook

Molecular electrocatalysts for CO_2_ reduction are of continuing interest for their possible utility in storing renewable energy in chemical bonds. Through careful mechanistic observations and design principles inspired by nature, researchers have managed to improve many of the known catalyst systems for CO_2_ reduction. This iterative optimization of catalysts has demonstrated that the incorporation of pendent Brønsted acids/bases, charged groups, sterically bulky groups, Lewis basic sites, and the use of multimetallic sites with careful solvent choice can lead to improved catalytic activity and even new mechanisms through secondary-sphere effects reminiscent of biological systems.

With these successes in mind, it is useful to acknowledge that there is still much work to do: CODHs can *reversibly* interconvert CO_2_/CO in aqueous systems with a TOF_max_ of 0.5 s^−1^ (CO_2_ to CO) and 31,000 s^−1^ (CO to CO_2_) with minimal overpotentials (Maynard and Lindahl, 2001). However, the use of secondary-sphere effects in abiotic molecular electrocatalysts has already been shown to be important for enhancing selectivity and activity. Further development in this area may lead to the development of catalysts capable of reducing products beyond CO and HCO_2_H, which are a burgeoning area for molecular electrocatalysts. The study of immobilized molecular Cu(TPP) systems has shown to be a successful strategy in generating higher ordered products like methane and ethylene in an electrocatalytic fashion (Weng et al., 2016), which is interesting, since crystalline copper electrodes are known to do this chemistry as heterogenous electrode materials (Kuhl et al., 2012). Bringing molecular design principles to materials seems to be another viable strategy for using secondary-sphere effects in electrocatalysis: Gong et al. (2017) immobilized porphyrin cages on Cu electrodes to tune activity and selectivity for carbon–carbon coupling products from CO_2_ reduction through supramolecular effects. Recent reports have also described photocatalysts which convert CO_2_ to methane from molecular catalysts related to those described here, which is promising for developing eventual electrocatalytic behavior (Rao et al., 2017; Shirley et al., 2019).

Running CO_2_ reduction reactions reversibly with abiotic systems could close the “loop” on the energy cycle, enabling the development of new fuel cell technologies beyond H_2_ (Matsumoto et al., 2011, 2013; Kadirov et al., 2018). New catalyst systems which approach these biological efficiencies are unlikely to be rapidly found through purely synthetic routes due to the inherent depth and synthetic difficulty of the parameter space. Rather, the most efficient approach to future catalyst development should utilize a multidisciplinary approach which combines statistical, computational, and experimental methods to assist in the search for new CO_2_ reduction catalysts unique from the current catalytic systems by improving predictive power.

## Author Contributions

The manuscript was written through contributions of all the authors. All authors have given approval to the final version of the manuscript.

### Conflict of Interest Statement

The authors declare that the research was conducted in the absence of any commercial or financial relationships that could be construed as a potential conflict of interest.
